# Implications of disparities in social and built environment antecedents to adult nature engagement

**DOI:** 10.1371/journal.pone.0274948

**Published:** 2022-09-23

**Authors:** Linda Powers Tomasso, Jose Guillermo Cedeño Laurent, Jarvis T. Chen, John D. Spengler

**Affiliations:** 1 Department of Environmental Health, Harvard T.H. Chan School of Public Health, Boston, MA, United States of America; 2 Population Health Sciences, Harvard University, Boston, MA, United States of America; 3 Department of Environmental and Occupational Health and Justice, Rutgers School of Public Health, Piscataway, NJ, United States of America; 4 Department of Social and Behavioral Sciences, Harvard T.H. Chan School of Public Health, Boston, MA, United States of America; Lorestan University, ISLAMIC REPUBLIC OF IRAN

## Abstract

Antecedent factors which influence adult engagement with nature are underexplored given the human health benefits strongly associated with nature exposure. Formative pathways and impediments to nature contact merit understanding as they may contribute to later-life health disparities. We probed experiential pathways and attitudes toward nature engagement among adults purposefully sampled across U.S. regions, age, race/ethnicity, and urbanicity through semi-structured focus group discussions. The research aims were to explore entryways and barriers to experiencing nature and learn how natured and built environments compete in influencing human-nature relationships. Sessions were recorded, transcribed, and analyzed following Braun and Clarke’s phases of thematic analysis. Qualitative content analysis of discussions identified three principal themes: 1) formative influences promoting adult nature engagement (i.e., persons/organizations and places of origin), 2) detractors from nature engagement (i.e., perceptual, material, and physical barriers), and 3) role of current setting (i.e., natural and built environments) shaping nature-seeking relationships. We found experiential factors that included early life exposures outdoors, personal mentorship, and organizational affiliation to be highly influential in socializing individuals to nature and in soldering attachment to nature which manifests into adulthood. In contrast, changing demographics and childhood, inequity, social dynamics, metropolitan growth, urban renewal explained alienation from nature. These findings emphasize the importance of efforts to expand opportunities for nature contact, especially for youth living in economically challenged urban areas, which go beyond increasing greenspace to encompass mentoring partnerships for gaining skills and comfort outdoors and redesign of safe natured spaces within cities for hands-on learning and discovery.

## Introduction

### Dimensions of nature exposure

Research documenting the health gains from nature exposure often concludes with the caveat that it remains unknown if and how individuals actually engage with nature [1–6]. Evidence from many countries suggests that population-based health endpoints generally improve with proximity, extent and appeal of urban greenspace and walkable infrastructure [7–11]. However, these mostly favorable outcomes reveal little of the social-structural antecedents which pre-position exposure levels fundamental to the nature-health relationship. What makes up nature exposure and how different subgroups regard and interact with nature remains ambiguous for gauging health targets. Who is being exposed to nature and under what conditions exposure occurs, if at all, requires greater attention as a population health [12] as well as environmental justice concern [13–16].

Time spent outdoors has declined for most children and adults globally [17–19]. For many, the built environment represents the only setting in which individuals live, work, and recreate, with urban greenspace the singular form of nature experienced by these individuals from childhood to adulthood [20–22]. Yet encountering safe, proximate, and useful greenspace can be problematic within the urban landscape [23, 24]. Metropolitan growth, building densification, and generational dispersal of families have changed nature interaction in spatially and socially determined ways, requiring effort to access natural spaces formerly more easily reached. Individuals thus feel more disengaged and alienated from routine nature experiences [25–27]. Nature alienation diminishes baseline knowledge of nature and its processes [28], biophilic attachment [29–34], pro-environmentalism [35–38], and even expectations of nature-based experiences [39]. Reduced nature exposure weakens opportunities to activate a latent nature affinity which predicts life-long nature contact and concern for environmental sustainability and stewardship [40–44].

The potential for experiencing the health benefits of nature contact furthermore remains beset by socioeconomic disparities [45–47]. Socioeconomic status (SES) is an acknowledged determinant of health as it shapes the likelihood and ability of adults to encounter supportive green environments for physical activity [48, 49] and mental health [50, 51]. In neighborhoods of low SES, greenspace not only is less available but often qualitatively inferior through poorer maintenance, less biodiversity, and fewer amenities [15, 52–54]. Vulnerable communities, frequently those of color, experience a form of environmental injustice [55–57] through exclusion from healthy nature contact [58]. Cross-cultural research shows children across income and race exhibit a universal biophilic concern; however, ecological degradation in socioeconomically deprived neighborhoods accentuates this concern, despite fewer tangible opportunities for nature interaction [59]. Such groundwork from child ecopsychology urges an exploration of social and environmental disparities upstream of nature contact as currently conceived and measured.

This study qualitatively explores nature engagement across race, region, age, and biophilic need to understand the origins of nature-seeking tendencies and reluctances to engage with nature that result in patterns of access and use. These methods are meant to complement empirical evidence on nature and health and reflect twenty-first century societal norms and patterns currently affecting historical human-nature relationships. Qualitative approaches seen in built environmental and health literature occasionally have addressed neighborhood-level factors, though not nature exposure per se. This research aims to 1) elucidate antecedent factors motivating nature-seeking in adulthood; 2) identify pertinent social and built environment contributors and barriers to nature use, which can amplify over time; and 3) discern elements common or unique to nature engagement or disengagement by specific group [60]. Our research objectives strongly bear on outcomes related to environmental health, given the potential for unaddressed nature deprivation to widen existing urban health disparities.

### Factors associated with nature engagement

A rich literature exploring predictors of environmental activism and ecological sensitivities points toward formative childhood experiences with nature [20, 61–66]. Significant life experience (SLE) anchors this research, with unfettered childhood discovery of the nature world, often in solitude, supporting the development of pro-environmental attitudes and activism in adulthood [58, 67–73]. Early SLE studies conducted among conservationists and environmental educations retroactively mapped childhood paths to known behavioral outcomes in adulthood [74, 75]. Far less is known about the motivational impulses behind nature-seeking outside these initial conservation-oriented professions. Moreover, the foundational SLE literature regarding nature engagement trails social and demographic transitions which pose constraints to current nature contact and predates the emergence of screen-based technologies, e.g., internet, social media, which crowd out time in nature [76, 77]. Metropolitan growth, urban densification, and declining car ownership additionally have made nature less accessible than when first formally examined [78].

Scales to measure nature connectedness have developed parallel to SLE work and help explain nature-seeking in adulthood [41, 43, 79–81]. Still, evidence that greater nature affinity favors the likelihood of pro-environmentalism does not shed light on the origins of nature affinity as a cultivated or innate sensibility, or correlated to trustful mentoring experiences, or specific to developmental age. Motivations to spend time in nature may be influenced in ways different from environmental protection, though both have implications for individual wellbeing as an extension of planetary health.

Current literature remains weak in addressing experiential precursors that set in motion different exposure pathways and desires to engage with nature across the life course [82], even though research indicates that social determinants related to urban nature access are spatially patterned [83, 84]. Local environments, population densities and sociodemographic attributes may exert different influences on individual access to and use of nature-based places not captured ecologically [85, 86]. Viewing the nature-health relationship in terms of antecedent natural, built, and social environments may unveil why adult individuals do and do not seek out nature beyond reasons of proximity. The ways different subpopulations “access, use, and respond to nature” therefore merit more exploration [4, 87].

Societal changes challenging nature use may embed factors that differentiate formative pathways culminating in active adult nature engagement. Thematic factors believed categorically relevant to nature relationships assemble around life events, operationalization, and emotions and cognitions. Life events consist of early life experiences in nature, influential adults who socialized participants to nature, and place-based associations with nature. Elements associated with operationalization of nature affinity regard effort to access nature, safety and wellbeing concerns, positive and negative emotions toward nature, inherited family environmental values, and knowledge of nature. Social attributes include age, mobility, organizational affiliation oriented to outdoor participation including schools, income by inference as facilitating or obstructing nature use, and perceived social exclusion. Factors in each of these categories are relevant to emotional imprinting, social vulnerability, and habit formation through early adulthood. Generational age provides a temporal lens to pick out ongoing levers of influence against those supplanted by fast-moving societal shifts which erode traditional pathways for discovering and partaking in nature. This research proposes to gain insights into experiential factors and socioenvironmental dynamics across varied demographic perspectives which enable and/or detract from a relationship empirically shown to improve individual health and wellbeing.

## Methods

### Theoretical approach to research design

We pursued phenomenology as our investigative method to explain the foundations of nature-seeking in adulthood through personal experiences of study participants [88]. Perspectives were inductively organized by inquiry aimed at describing the semantic meaning and significance of personally narrated events [89, 90]. The use of focus group discussions (FGDs) was well suited to a research design reliant on ancillary organizers to recruit and convene study participants unaffiliated with the interviewer [91, 92]. COREQ reporting guidelines helped frame and report this study to improve transparency and analytical rigor of interview design and analysis [90].

### Participant recruitment

Our study population consisted of individuals recruited in two waves to explore formative experiences and origins of attitudes shaping nature-seeking behaviors as adults. Recruitment initiated with Facebook advertisements placed October 2019 in four geographically diverse regions inviting volunteers to an on-line enrollment portal explaining study goals. Inclusion criteria included over-18 adult age and ability to attend an in-person focus group in one of the four target areas. 596 participants registered through the on-line portal, listing only city and gender, which resulted in a meta-population of 82.8% women, 15.0% men, 1.5% non-binary gender. The goal of gender parity meant all enrollees who identified as male or non-binary were invited by email to attend a focus group, with rolling replacement invitations issued to females until twelve participants per group confirmed. Males responded at higher rates but had lower attendance availability, while females had opposite response trends. Having discovered that variation sampling strategy [93] did not yield adequate demographic representation necessary to conduct this study, we sought to expand sampling representation through an affiliated organizational network of public health fellows who work with local community groups. We then invited community group members who might bring economic, racial, gender and age diversity to our sampling population. This second recruitment method allowed us to pair one Facebook-recruited focus group comprised of varied but self-selected participants from each metro area with a more homogeneous focus group defined by sociodemographic dimensions underrepresented through ad response. The addition of two conservation groups provided a subpopulation comparable to those studied in the original SLE literature and which also bookended the spectrum of participant time spent in nature. All final study participants voluntarily enrolled and provided electronic consent.

### Study setting

We convened study participants from the environs of San Francisco Bay, Atlanta, Phoenix, Boston, and Hartford and organized a session for conservation officers at their annual national conference. All FGDs were conducted in-person at a community meeting room or university classroom except for a final discussion held over Zoom in accordance with IRB protocol restrictions under COVID-19.

### Participatory research tools

The research team chose FGDs for efficiency over individual interviews. A semi-structured topic guide (S1 Table) informed by the background literature [61, 67, 94, 95] and expert recommendations for future nature-health research [87] was developed specifically to explore individuals’ attitudes and experiences which shape current nature engagement, refined for participant inclusivity. An introduction to the research purpose began each session. Participants were next invited to recall a place meaningfully associated with nature and reasons supporting their choice to establish the thematic arc of the topic guide. New subtopics which arose were incorporated into subsequent sessions owing to the iterative nature of the research methods [96].

### Ethics statement

All participants reiterated oral consent prior to commencing the discussion and were informed of their right to opt out at any point during the process. The study was conducted according to the guidelines of the Declaration of Helsinki and approved by the Institutional Review Board of Harvard T.H. Chan School of Public Health (protocol code 19–1419 on 28 August 2019). Arizona and Georgia State Universities additionally consented to allow data collection under the referenced IRB.

### Data collection

The first author and main facilitator (LPT) conducted ten FGDs lasting between 1.5 to two hours each, with 8–19 participants per group. Data were collected in-person between September 2019 to January 2020 and over Zoom in November 2020. The sessions were digitally recorded with participants’ permission, transcribed verbatim by an outside person, and reviewed by the interviewer to ensure accuracy. Transcripts contained no identifiable participant information or self-identified sociodemographic data beyond gender and region gathered at study enrollment. Theoretical saturation was determined once no new themes surfaced in ongoing data analysis, suggesting adequate sampling representation [90]. Focus group participants received a $20 retail gift card at session conclusion to acknowledge their time.

### Data analysis

**Fig 1:**
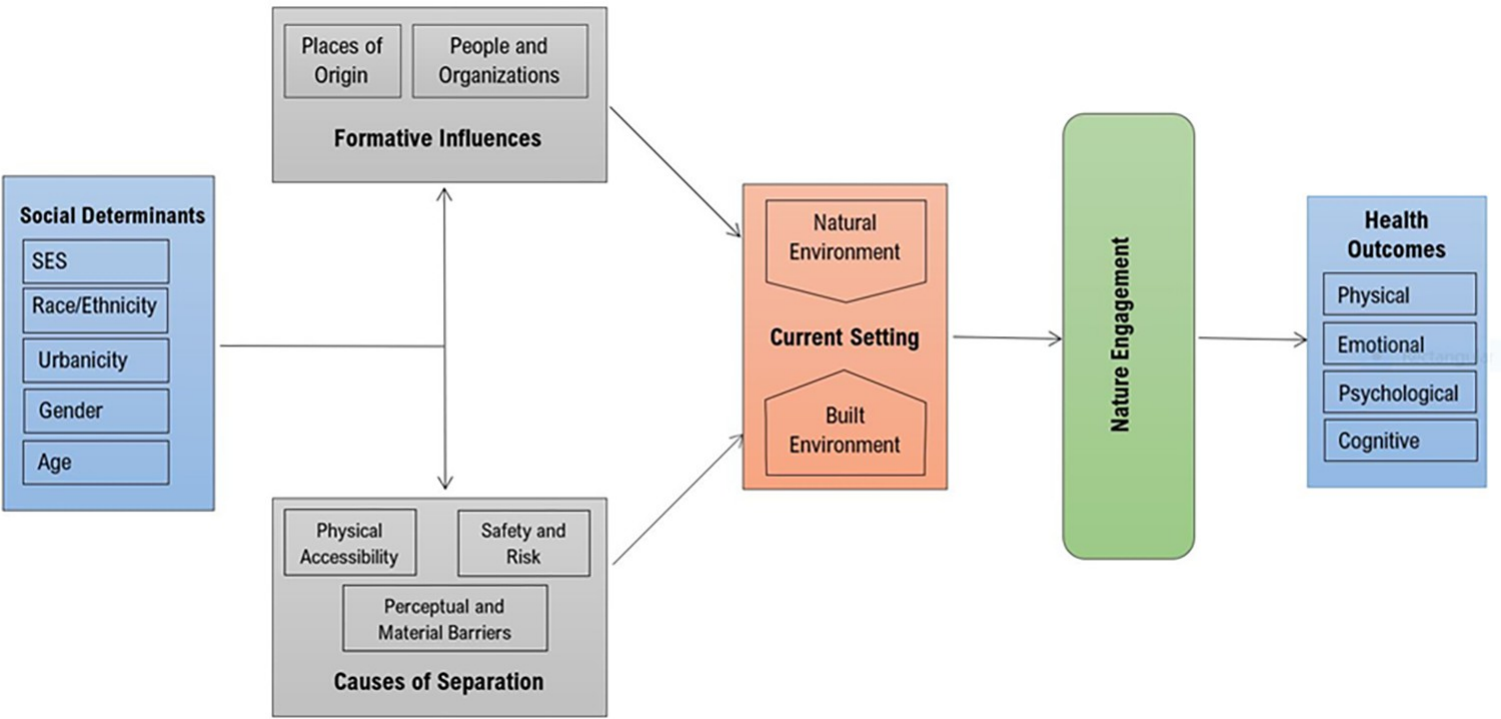
Conceptual framework describing a general model of exposure pathways to nature engagement identified through qualitative methods.

Transcribed interview content was analyzed and managed using NVivo 12 Plus [97]. After transcript review, the FGD facilitator (LPT) inductively identified codes based on subject knowledge and group interface. LPT subsequently developed a code manual to define emergent themes and subthemes based on the study framework and existing literature, specifying when and when not to use each [98], to share with the second analyst (MMF). MMF identified further subthemes not covered by the first analyst (LPT) to produce a final codebook. Open coding allowed the two analysts to ascribe additional subthemes to the initial organizational scheme when omissions and new ideas were detected [99] until consensus was reached on themes. The analysis was performed systematically across the ten groups and independently of the study guide questions. The study’s internal validity was strengthened through analysts’ regular discussions of contextual meaning based on increasing familiarity with transcript content and participant intention. Inter-rater reliability was maintained through multiple and separate reading of transcripts to reconcile coding discrepancies and mitigate any bias that may occur during data interpretation, merge overlapping subthemes, and drop nascent themes where text evidence proved insufficient [92]. LPT created a concept map depicted as Fig 1 to connect final categories as supported by thematic evidence [96, 99–101]. Together the two analysts coded a total of 61.5% of the transcript data, reaching concordance on 82.6% [102, 103]. An unweighted kappa coefficient of 78.3% measured agreement corrected for chance.

## Results

**Fig 2:**
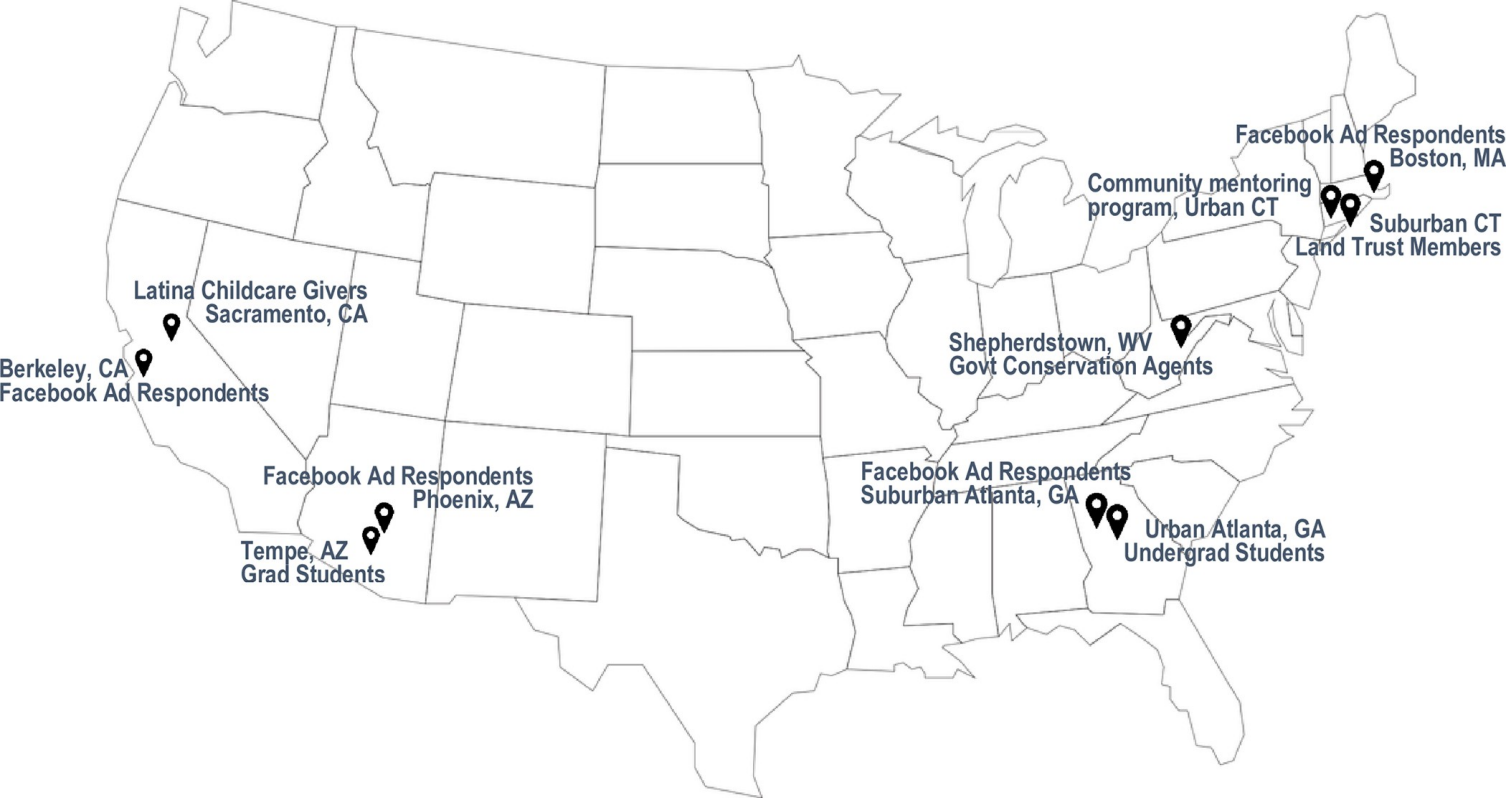
Focus group locations. Ten focus groups were held in four metropolitan regions and at a national conference of federal conservation officials. U.S. map is made available under the Creative Commons CC0 1.0 Universal Public Domain Dedication.

Our 127 participants represented 49 individuals recruited through variation sampling, 44 through an affiliated public health network, and 34 professionally or avocationally working in conservation. The four ad respondent groups were of mixed demographics, the community network groups of homogenous character by race/ethnicity and/or age, and the conservation groups likewise homogenous in terms of race, suburban or rural residence, and slightly older mean age. Two groups were predominantly composed of black individuals, and one group of Central and South American origin. This latter FGD was conducted in Spanish. Persons of color participated in some of the Facebook response groups, bringing representation of non-white individuals to one-third of total study population. Lower income groups based on recruitment census tract represented one-quarter of participants. Table 1 describes focus group characteristics; Fig 2 illustrates focus group locations.

**Table 1:** Focus groups characteristics.

Group	Location	Recruitment type	Gender^b^	Age Range	N =
1	Tempe, AZ	PH^a^ community network	6 F, 5 M	25–35	11
2	Phoenix	Facebook ad respondents	4 F, 6 M	Mixed age	10
3	Rural WV	Govt conservation officials	5 F, 10 M	40–65	15
4	Boston, MA	Facebook ad respondents	11 F, 3 M, 1 NB	Mixed age	15
5	Urban CT	PH community network	2 F, 8 M	18–22	10
6	Downtown Atlanta	PH community network	13 F, 2 M	18–22	15
7	Suburban Atlanta	Facebook ad respondents	9 F, 2 M	Mixed age	11
8	Berkeley, CA	Facebook ad respondents	9 F, 3 M, 1 NB	Mixed age	13
9	Sacramento, CA	PH community network	7 F, 1 M	24–28	8
10	Suburban CT	Local conservation group	11 F, 8 M	Over 50	19

^a^ PH = public health

^b^ M = male, F = female, NB = Non-binary

### What formative influence shape our affinity for nature?

Formative influences, grouped as places of origin and persons or organizations providing positive introductions to nature, strongly inclined individuals to pursue nature engagement in adulthood (S2 and S3 Tables).

#### Formative places

Place of origin conditioned life-long attachments to regional geographies independent of current setting. Growing up near untamed nature enabled free exploration outdoors which shaped lifelong affinity for wildness. Childhood vacations awakened nature affinity and imparted assurance and skills to engage comfortably in nature for many.

*I’m from Madison*, *Wisconsin*. *We grew up going to state parks a lot*. *Madison used to have more parks per capita than any other place in the country*. *We were always going to parks*, *whether city parks in Madison or the state parks*. *One in particular*, *Devil’s Lake State Park*, *has a lot of glacial features*, *tumbled rocks*, *so that’s my favorite place to be*. Suburban Atlanta, Family travel subtheme

Participants, particularly those in conservation and those foreign-born, described deeply held emotions toward favorite places and memories formed there [104]. Interviewees frequently mentioned discovery of new landscapes in adulthood which cast childhood nature experiences as different and unique, a realization also common to those from less-natured areas. Participants over-50 spoke fondly of developing nature affinity through unsupervised play outdoors. In contrast, younger interviewees and those raised in large cities rarely played outdoors unattended.

*The closest I got to nature was when I went to ‘Cali’ and I hiked the Hollywood Sign*, *all the way to the top*. *That’s when I really experienced the way nature is and seeing how it’s different from Connecticut*. *Just being way at the top*, *it literally hit me*, *like wow*. *It’s a really different life from here to there*. Urban CT. Uniqueness of own nature subtheme

#### Formative persons and organizations

Participants very specifically recalled persons or organizations responsible for “socializing” them to nature. Women and men post-45 credited fathers for introducing them to nature via hands-on knowledge-sharing in reassuring natural environments. Busy mothers played direct and indirect roles as nature advocates, spearheading outdoor activities or banishing children outside. Under-30 individuals infrequently mentioned their parents or family members having introduced them to nature, except those foreign-born, particularly Afro-Caribbean. Individuals from all demographics said that nearby older relatives, particularly grandparents, strongly shaped their childhood experiences in nature. Transfer of ecological values through vegetable gardening, growing seasons, and plant hardiness was intrinsic to time spent in nature with grandparents who metaphorically conveyed life lessons of self-reliance under adversity.

*A significant person would have to be my great-grandmother and my grandmother*. *Growing up*, *they had their own houses down in Grenada and their own farmland where they produced their own food products*. *So it was definitely a wonderful experience just watching natural things grow*. Downtown Atlanta. Extended family subtheme

Camp played an oversized role for city kids with little family or backyard introduction to nature. Urban participants of all ages used rich, sensory language to recall memories of bonfires, night skies, and tent camping discovered at overnight camp. U.S. and international participants spoke of acquiring high skill levels and comfort outdoors through scouting or church excursions. Urban residents with weaker institutional support mentioned extracurricular athletics, e.g., school football, as their nature introduction.

*In middle school I was part of a church-sponsored ecology group*. *One of our best trips was riding our bikes from Atlanta to St*. *Simon’s Island which took us five days*. *We had a chaperone group that would go ahead of us and set up tents and then mark our spots*. *That was the most influential*, *to be part of those groups*. Suburban Atlanta. Scouts/organization subtheme

### How does environment influence nature engagement?

Discussants spoke of ways current settings have unwittingly bolstered a desire for nature engagement by pulling toward natural environments and pushing against built environments (S4 and S5 Tables).

#### Push of built environments

Many speakers referenced nature as antidotal to built environments, which they associated with frustration and entrapment. Repudiation of indoor environments even opened some conservation rangers’ permanent career paths. Nearly all participants found the “artifice” of anything manufactured to detract from nature enjoyment, though urbanites less stridently opposed constructed facilities. While suburban, retired, and non-working individuals seemed less agitated by structural enclosures, office workers described turning to nature-based recreation to escape the pressures of current work.

*My job is one of those twenty-hours-a-day*, *you’re never not working*, *always answering emails and phone calls and everything*. *So we started purposely planning trips—hey*, *we’re going to hike through the Smokies or the Tetons*, *go someplace where my phone’s not going to work*. *So getting away is my touchstone to reset and rediscover what’s actually a priority*. New Jersey. Nature as place of escape

Encountering other people outdoors generated mixed emotions, though many participants rebuffed venues supporting elevated levels of visitation as “nature.”

*It added something for me when people were [visible] rather than only the environment*. Minnesota

*Now I’m just the opposite*. *For me*, *I want to be by myself in nature*. California. Human-nature interaction subtheme

#### Pull of natural environments

Geographies experienced early in life appeared permanently to imprint participants’ notion of nature. Places lived and experienced caused many to feel proprietary toward specific landscapes, perhaps revealing regional bias, and influenced a dissatisfaction toward current nature availability for some. Individuals in the two land-locked sites repeatedly mentioned yearning for waterscapes. Backyard gardens provided private nature enclaves for some urban residents uninterested in expending greater effort to scale-up landscape exposure. Other city dwellers highlighted the unexpected delights of urban nature.

*What really brought me a lot of joy as I got older was my first real trip on my own to Washington D*.*C*. *I was down there during the season of cherry blossoms*, *and—even though I still think of D*.*C*. *as a city–just having nature in the middle of the city… it’s so peaceful walking there at night*, *and it was also a calm*, *soothing and beautiful experience*. Urban CT. Pull of natural environment subtheme

### What separates us from nature engagement?

Obstacles to nature engagement fell into three general categories: individual-level material or perceptual barriers, public-level physical and structural barriers, and safety concerns (S6–S8 Tables).

#### Perceptual and material barriers

Lack of time topped every focus group’s recitation of barriers to nature access. Consuming weekday and weekend schedules of work, college studies, and child-raising leave little free time for leisure of any type, including nature. Subjective hurdles, e.g., guilt, lax motivation, or preference for indoor comforts, further interfere with routine nature contact. Perceived social exclusion, marginalization vis-à-vis other greenspace users, or behavioral expectations limited enjoyment of nature sites for some urban individuals of color, who also expressed frustration over restrictive use of city parks. Some participants mentioned feeling uncomfortable and unsure around nature due to a lack of mentorship which stood in their way of accessing and enjoying nature. Mothers in attendance conveyed how parenting pressures, even in two-earner families, compromise their ability to bring children into nature in ways they remember from childhood. Central and South American childcare workers sharply noted an indifference toward nature within U.S. families vis-à-vis their homelands that is reinforced by working parents too fatigued to plan outdoor excursions on weekends and young children’s captivation with technology [105, 106]. Technology was reviled as directly competing with time outdoors and intrusively disrupting nature’s quiet, though a few praised app-based technologies for making nature excursions more accessible and time efficient. Nonetheless, participants of every age blamed generational changes underlying nature withdrawal.

*I have 17 great-nieces and -nephews*, *and they all come up and spend time in eastern Washington*. *Richard Louv had released “Last Child in the Woods*,*” and I tried to see if that really mattered regarding nature and behavioral issues*. *We arrived on Thursday evening and didn’t go back until Sunday*. *And the difference from when they got there and when they left was absolutely measurable and marked*. *It was incredible*, *so wonderful to see that being facilitated by nature*. Phoenix. Generational differences subtheme

Finances and vehicle ownership posed the principal material obstacles to ex-urban nature engagement for many younger individuals. Camping vacations created a generational fault line drawn by financially out-of-reach equipment and transport. Older commenters amusingly recalled family “budget” camping trips from childhood, while younger parents winced at a family camping vacation’s prohibitive cost. Older individuals with fewer barriers to nature access—a primary or second residence outside urban cores near nature, car ownership, and some financial cushion—appeared more resolved to spend as much time outdoors prior to ill health’s onset.

#### Physical and structural barriers

Respondents generally viewed nature access as a function of convenience and preference. Most participants except core city residents felt urban nature did not suffice if wild nature lay within a 1.5 to 2-hour car ride. A few suburban participants spoke of satisfying biophilic need locally given the time and transport required for large-scale nature access. City dwellers from sparsely vegetated neighborhoods interpreted nature contact more liberally, as infrastructure shortcomings preclude them from fuller nature exposure. Inefficient transit systems and sidewalk disrepair discouraged many residents of larger cities such as Atlanta even to attempt getting outdoors.

*I’ve tried to go to the parks with the waterfall*. *They have certain times that they’re open*. *You have to plan to be there early so you can be there long enough—if you do have to drive or travel—kind of to make that trip worth it*. Urban Atlanta. Barriers to nature subtheme

#### Safety and risk concerns

Safety perceptions and risk aversion presented further wedges to nature-seeking. Focus group members premised their responses around previous outdoor experiences, finding the question “is nature safe?” highly subjective. Some contextualized safety to one’s personal location. Others discriminated safety from comfort, focusing on risk management in nature to stave off potentially unsafe situations. While most agreed that feeling safe in nature is tantamount for enjoying the outdoors, group-level dynamics established a spectrum of safety bounded by instinctive comfort and unnecessary risk-taking [107]. A few men also remarked on the potential for nature’s destructiveness for fear of property damage. Several commenters found risk-taking inherent to nature contact and risk key to nature’s attractiveness. A few male and female Southwesterners recounted harrowing episodes in extreme nature, concluding that the high stakes nature poses created a sense of vulnerability that had permanently shaped their character.

*The memory of the creek was a place that I wanted to be because it was unsafe*. *It wasn’t super unsafe*, *but there was a sort of wildness and a fierce independence in being in that space*, *particularly as a kid all by myself*. *But that was somehow part of the allure for me*. Tempe, AZ. Thrill of pushing safety boundaries subtheme

All groups debated if people or nature posed the greater threat outdoors, though “creepy people” were commonly perceived as the greater endangerment. Inexperience in nature paired with feeling safer near crowds; conversely, interviewees comfortable outdoors admitted to high urban anxiety.

*The fact that it’s a safe nature space is calming enough for me that no matter what dangers there are*, *whether animal or environmental*, *it’s still going to feel safer than what may happen in an unnatural space like a city*. Berkeley. Nature vs human safety subtheme

We found alignment of thought along demographic attribute regarding issues of safety. Non-urban females emphasized feeling more secure in nature than they do within built environments. Urban residents of color, male and female alike, spoke of nature as a forbidding, fearful place. Participants of all races expressed concern over racial and SES foundations of inequitable nature access, particularly for children.

*Probably 3% of students are white*. *Most are black and Hispanic and a few Asians*. *They don’t go outside*. *Many live in Section 8 housing*. *A lot of my students are not permitted to go outside because their families worry about their safety*. *So they never*, *ever get outside*. *Often*, *they don’t even feel safe inside their own homes*, *much less just in the yard*. Suburban Atlanta. Equity subtheme

One non-white participant recounted perceived prejudice while hiking, adding that more rangers and trail-users of color becoming a familiar site could diffuse reciprocal outdoor unease.

*Most of my experiences in nature are very positive because I create them*, *but they’re not always positive because people project a sense of fear when I’m approaching*. *So I feel safe*, *but people may not always feel safe around me*. *So seeing a park ranger who looks like me makes the difference*. *Seeing another person who is not white in nature is important*. Boston. Safety and risk/ Equity subthemes

## Discussion

We approached nature contact as experienced outdoors and explored through qualitative methods how individuals form perceptions and patterns around their own nature use. Inductive thematic analysis across ten focus group sessions showed a concurrent, dialectical pull of early life influences toward nature in tension with multiple barriers pushing individuals away from adult nature engagement. Most but not all study participants spoke of spending time outdoors. In examining current social complexities underpinning nature exposure and nature equity, our discussions revealed how upstream factors predispose individuals to engage or disengage with nature early in life and subsequently perpetuate disparities in nature contact into adulthood. Data organization and analysis across a variety of geographies, population densities, and sociodemographics uncovered a clear patterning around the bases for engaging in nature and the hazards or barriers to accessing nature in ways not entirely predicted at study onset. We found a decisive cluster of factors common and recurring across subpopulations which incline individuals toward adult nature-seeking, along with uniquenesses defying prevalent trends.

In seeking to elucidate antecedent factors motivating nature-seeking in adulthood, our first study objective, we found two distinct but consistent models of entry into nature for most individuals. The first model appears to set in motion deep-seated emotional attachments to nature and intentional pursuit of familiar natured environments and is heavily and positively characterized by childhood place of origin, trusted adult mentorship, and current setting merging supportive built and natural environments. Some acute and chronic barriers detract from lifetime nature engagement, e.g., urban densities, distance to nature in childhood, but remain surmountable given resource availability and personal interest.

Central to this model for most individuals over-30 who self-described as currently active outdoors was socialization to nature in childhood through adult modeling, supplemented by organizations when family and neighborhood context waived that role [108]. Many conservationists and Facebook ad respondents fit this profile. Concurrence across our study reaffirms the importance of mentorship in socializing children to nature [63, 109, 110]. Without direct nature interaction guided by knowledgeable mentors, children may lose an important connection to inherent sensibilities [66]. Previous research has found that adult support of children’s nature-based play predicts heightened self-discovery [111, 112], deeper nature affinity [41], and more complex psychological, emotional, and physical development than what green exercise alone offers [113], despite some evidence to the contrary [110, 114].

Detailed thematic analysis also revealed that place of origin strongly primes individuals toward nature engagement by shaping an interconnected cultural set centered around childhood residence, family vacation sites, special natured corners for unsupervised play, and nature proximity in childhood. Place of origin showed robust imprinting on participants’ positive attitudes toward nature and their continued desire for nature contact as adults, despite an oft-greater effort to access natural environments resembling those frequented in childhood. A geography of childhood tied to contact with wild nature overshadowed current setting in drawing individuals outdoors [115–117]. Individuals introduced to nature in childhood make deliberate choices as adults to seek out nature despite constraints like time [118, 119]. In fact, recollection of early imprinted nature stirred up feelings of separation in participants who differentiated currently accessible nature from “the nature I desire.”

Such findings reaffirm existing literature on place-based identity. Not only does place shape the experiences that contribute to identity formation [115], along with attitudes, beliefs and behaviors toward nature [69], but nature availability in childhood reinforces a sense of place and wellbeing created through lasting experiences in untamed natural environments [120]. Nature-centered play in childhood is shown to exceed all other background variables in informing frequency of adult time in nature, with weak childhood nature contact predicting low or no adult nature visitation [113, 121, 122]. Most adults, including our study participants, identify the outdoors as their most significant childhood place such that the landscapes of childhood serve not just as backdrop to other events, but memorable events unto themselves [123].

An alternative nature entry model characterizes urban, socioeconomically challenged groups, resulting in more circumstantial, passive nature contact and cautioned use of natured environments. Reduced opportunities for formative learning and engagement in nature at a younger age, fewer nature-inclined mentors, layered spatial and structural barriers to access nature, and urban inefficiencies factor more heavily within this paradigm. Negative perceptions of wild nature also off-put individuals lacking reassuring introduction to untamed nature [123]. Opportunities to spend time in wild nature have worsened for this population as extended family members sell off small family farms and rural homes for urban locations, creating more insular urban nature contact. Early-life impediments to experiencing nature open pathways to adult nature alienation and forfeit the potential for health-inducing nature exposure.

Our second study aim of identifying pertinent social and built environment contributors and barriers to current nature use found many common roots of nature disengagement across our study population and fewer shared elements predicting nature engagement. Most individuals attributed their nature separation to social reasons as varied as time pressures, workloads, and competing attractions. The universal curse of scarce time imposed by work, childcare, and weekend chores affecting all participants echoes previous findings on time infringements on nature engagement [124]. Individuals today not only have less time overall for nature engagement but for recreation in general. Resource barriers to equitably accessing nature tended to focus on lack of money for equipment or user fees, though income elasticity only partially explained why certain groups do not partake in recreational nature pursuits. Transit inadequacy, for instance, stymies pursuit of larger landscapes specific to nature-based activities such as hiking, cutting off options for vigorous physical activity supporting health and wellbeing for many urbanites who cannot or choose not to acquire a car.

Societal transformations of the past two generations have additionally compounded adult nature disengagement in ways few individuals are immune. First among these is the changed nature of childhood. Harried parents a generation ago “threw [kids] out into nature;” today, busy parents reluctantly keep children inside due to public safety concerns. As compared with prior generations, often children today have more structured, activity-based schedules and less overall free time to spend meandering outdoors without parental oversight. Parents who allow children to play unsupervised outside have even been accused of negligence [125]. Children in day- or afterschool- care at best may have highly circumscribed on-site outdoor time, while within the home even young children’s absorption with electronics and video games negates actual nature beyond their screens. The withdrawal of independent childhood play in nature has diminished opportunities to explore outdoors and with them, sensory capacities developed from interacting with biodiversity [126]. Moreover, affordances for creative nature-based play on indistinct urban greenspace dim when compared to more organic, wild spaces [127].

A second societal transformation concerns the weakening of family and organizational structures which has negatively impacted traditional mentoring relationships outdoors, the most cited contributor to nature engagement. Declines in membership groups like scouting, civic and religious affiliation, and local business patronage have meant reduced operational and financial support of community- or church-run summer nature camps, jeopardizing a positive and perhaps one-time experience in wild nature which particularly impacts urban youth. The loss of wild nature contact in childhood is transversal: *“I was a boy scout growing up*, *so I got a lot of merit badges for nature*, *so my experience was right there*.*”* Such generationally-rooted observations suggest a gradual alienation from untamed nature felt by many adults and most acutely by young urban residents, with the potential for deepening health disparities posited on the benefits of early outdoor exposure [128, 129].

Feeling safe in nature also manifested as a learned skill which deters many urban participants not acculturated to nature over time. Outdoor phobias such as fear of snakes and bears [130, 131], entomophobic disgust, or dread of nightfall are confronted and overcome through social modeling, while differences in the physical structuring of natural environments influence perceived safety, e.g., disorientation [132, 133]. Woodlands offer low degrees of prospect and refuge [134] so that evolutionary response combined with cultural legacy may heighten urban non-white residents’ distrust of forested environments. Awareness and management of risk is therefore central to outdoor mentorship. Our results stand up to previous research findings in the older SLE literature that mentorship in nature is critical in operationalizing long-term nature attachment and its use.

Assumptions of the nature-health relationship are heavily premised on the spatial patterning of urban nature access where socioeconomic factors of park use are embedded but not made explicit. Greenspace proximity disregards neighborhood safety—even backyard safety—which effectually renders many urban parks inaccessible. Young urban adults we spoke with confirmed they avoid local greenspace when connector neighborhoods feel unsafe, regardless of security enhancements made within parks. They similarly fear strangers who frequent local parks, a phenomenon not previously observed in move vegetated neighborhoods [135]. While many lifelong city residents favorably embraced urban ecology broadly described, young adults of color spoke apprehensively of urban forest environments. Research evidence both upholds and refutes this last observation [53, 132].

The third study objective aimed to discern elements common or unique to nature engagement or disengagement by specific group. Access to natured areas within and beyond city limits depends on functional built environments, though sprawl, transit inadequacy, and concentrated urban green space discouraged nature pursuit for many. Furthermore, these built environment changes interact with and compound one other. For example, expanded urban districts demand more time to reach landscape-scale nature, so the value proposition of spending time in nature diminishes, even for those owning a car. This spatial reality suggests that urban residents craving nature’s restorative offerings face less surmountable barriers to access healthful natured environments than residents of lower-density areas.

Despite some occurrences of racial stigmatization, urban young adults felt drawn to city parks more than big landscape, provided urban greenspace offers “something useful to do.” Equalizing park access and quality to promote health of urban residents extends this mandate [128], but urban greenspace does not in itself give children the affordances and opportunities for independent nature discovery. In fact, much greenspace viewed aerially consists of baseball diamonds or cemeteries, neither being of much everyday use to most individuals nor can feel more artificial than natural. This observation speaks to informal park amenities: if children lack safe and receptive places to interact with nature, their nature contact remains purely visual. At the same time, urban recreational greenspace offers scant opportunity for the solitude and emotional reset so highly prized by landscape-scale nature-seekers but off-limits in areas where personal safety concerns eclipse the need for urban stress reduction.

Three insights differentiate this research from existing scholarship: 1. Dual spatial contexts—the neighborhood and the city itself—mark urban nature accessibility. Neighborhood context is determined by nature proximity, vegetation amount and quality, and “safe passage” to greenspace. The city context is proxied by the effort individuals can expend in leaving the built environment to reach their defined nature, with its opportunities for active recreation, solitude, and mental restoration. 2. Dual temporal contexts of childhood formative experiences in nearby nature that once timelessly held a child’s attention have now ceded to inside play, activity scheduling, safety concerns, and screentime fascination, diminishing children’s independent nature experiences which seed healthful adult associations outdoors. 3. The retreat of close family members and other mentors who once provided hands-on interaction in nature by positively introducing and modeling outdoor acculturation, thus inspiring curiosity as well as resilience outdoors. Teachers and schools may fill that role today if time, inclination, and nature proximity permit, but such convergence remains rare in high-density areas. Taken together, this trio of spatial, temporal, and social factors reveals widening structural differences between city and non-city dwellers and that put the human health-nature relationship at risk, particularly for young residents of low-income urban areas.

### Implications and recommendations

We detected three groups of individuals whose possibilities for nature engagement cluster spatially: 1. those for whom access to nature is impeded by neighborhood, including safety; 2. those deterred from active nature recreation by urban density and cost. 3. those fully able to partake in close landscape-scale nature, restricted only by time. This latter group derives a clearly articulated set of benefits from the nature experience discernible as mental and emotional wellbeing linked to solitude in nature, physical activity specific to outdoor nature sites, and a sense of accomplishment achieved through goals oriented to nature. Users of urban greenspace, despite being in nature, rarely spoke of these benefits. Park revitalization might mean offering safe spaces for contemplative reflection and “something to do” on greenspace like ropes courses, adventure education, or urban forest loops so that urban residents can choose relaxation with the self-satisfaction that comes with goals achieved as a form of positive risk-reward. The thrill of pushing boundaries and feelings of victory instrumental to outdoor enjoyment embody positive psychological outcomes linked to nature-based adventure sports [136].

Public policy lags science concerning the conservation and use of urban natural environments to achieve health objectives. In addition to furnishing ecological benefits [137], urban greenspace associates with improvements in neurocognitive functioning to impact behavior [138]. Studies from various global cities confirm that prioritizing biodiversity in planning urban greenspace yields improvements in mental health [139, 140] and emotional regulation [141]. In Berlin, the amount of forest coverage was associated with greater amygdala integrity, suggesting that certain forms of biodiversity natured environments positively influence brain plasticity [142]. Ongoing gaps in public policy suggest the undervaluation of urban vegetation to support the cognitive health and emotional wellbeing of individuals including the aging [143].

Recommendations to “design communities that facilitate free access to nature” are not novel and expressly stem from earlier investigation of how conservation behaviors develop among children and adolescents [111]. Further research is needed to learn if interventions for children to experience urban nature through creative, haptic play can emulate nature engagement in lower-density environments where geographies of childhood were formerly cemented. Affordances for creative nature-based play on indistinct urban greenspace dim when compared to more organic, wild spaces [127]. Studies investigating prescriptive nature contact should consider the role of mentorship in teaching urban youth how to engage safely and confidently with nature in both wild and urban settings. Nature exposure alone may not suffice if contextual support for socializing children to nature is missing [144].

As a future next direction we recommend participatory research bringing together stakeholders whose inputs for designing, using, and managing of urban nature inform improvements of the city park experience. Participants could include municipal managers of parks and recreations, public health, youth and senior service departments, public works officials to discuss safe corridors to access parks, nature users of various ages and interests, and community not-for-profits to operationalize nature use. Municipal parks departments have observed facility under-utilization. Park non-use speaks to the need for programmatic services, not just greenspace. What should urban parks offer families, adolescents, older adults to engage and relax? Focus groups can present place-making of different nature scenarios, e.g., water features like riverine or pond trails, exploratory sites for children, or immersive, pristine parcels such as Boston’s Urban Wilds program, to create preferences for nature environments where people will want to develop recurrent and habitual engagement with nature.

### Strengths

Our multi-site study design and access to a range of perspectives into nature use offered original insights on social and structural factors of nature contact absent from quantitative methods. Multi-site research importantly provided a broader foundation for discovering patterns between sites or among subsets of sites and greater validity for drawing conclusions than single-site inquiries allow [145, 146]. The influence of diverse regional culture, climate and geography on participant perspectives strengthened opportunities to discover similarities in attitudes and practices of using nature which transcend place and circumstance while accentuating uniquenesses tied to individual origin and on-going experience. Qualitative data collection occurred across heterogeneous groups, e.g., Facebook ad respondents, and more homogeneous groups, e.g., liaised community networks. The variability of participant experiences introduced into the nature and health literature responds to recommendations to include individual factors into ecological studies.

### Limitations

Our findings should be considered in light of study limitations. A population sampling strategy that relied on advertised recruitment and self-selection likely limited prospective participation; older mean participant age perhaps owed to FGD scheduling availability and use of Facebook Timeline less popular with younger audiences. Data collected may reflect an inability to have reached all individual viewpoints due to research methods aimed at group engagement. While we sought out the broadest socio-demographic range available and engageable to describe nature experiences, we were unable to obtain viewpoints most likely to reflect nature disenfranchisement. The community networked focus groups of urban young adults made up the more economically challenged groups, making us unable to associate SES with nature engagement at later life-course stages. The Facebook ad respondent groups may have included individuals across the socioeconomic spectrum, but since we did not capture individual-level characteristics at intake beyond gender and region, we could only infer participant attributes from commentary. Our imbalanced gender ratio in many focus groups may have inhibited deeper narratives tied to gender in operationalizing nature contact as well as an under-exploration of gender vis-à-vis the nature construct. In-person attendance requirement and meeting times likely deterred families, married couples, and hourly workers from participating, depriving our study of important demographic perspectives of affordability and safety in nature outside individualized experience.

## Conclusion

In this article, we presented results from our investigation of antecedents to adult nature engagement as a qualitative extension of exposure assessment. Our qualitative inquiry sought to complement statistical findings around exposure patterning by examining current social complexities underpinning nature use. We methodically gathered and studied upstream contributors which shape adult inclinations and abilities to engage in nature.

We found that few influences substitute for direct outdoor experience in childhood, personal mentorship to acquire comfort and skill in nature, and/or organizational affiliation, particular for children raised in dense built environments, to become socialized to nature. These factors featured across subpopulations and sociodemographic attributes. Individuals today face a concurrent suite of barriers to nature immersion that includes time, urbanization, job responsibilities, uncertain quality of nature experiences, transportation, lack of know-how in nature, and, for many, costs associated with nature engagement [147]. The changing nature of childhood may truthfully predict why many children no longer play independently outdoors as much as distance to greenspace or recreational affordances within these spaces does [76, 148, 149]. Greater time constraints and safety worries faced by working parents translate into less park use for children in city cores vs. suburban communities [150]. These obstacles afford fewer opportunities to expose children to nature than available in previous generations.

In obtaining the views of a broad population toward nature engagement, this research presents perspectives and experiences useful to developing means for surmounting challenges to nature disengagement which are disproportionately distributed due to social and environmental factors. Understanding what enables and detracts from nature-seeking attitudes and behaviors grounds intervention options. Identifying patterns in nature engagement across regions and subpopulations and uniquenesses within them can inform integrated public health policy and urban planning decisions around meaningful nature access at younger ages. Equitable as well as healthful nature engagement antecedents to measured exposures can then carry the nature-health paradigm toward fuller fruition.

## Supporting information

S1 Table Topic guide for semi-structured focus group discussion. (DOCX)

S2 Table Formative places in nature. Selected participant comments share how formative places in nature in childhood influenced adult nature engagement.
(DOCX)

S3 Table Formative persons and organizations. Selected participant comments discuss formative persons or organizations who socialized them to nature in childhood.
(DOCX)

S4 Table Push of the built environment. Selected comments reveal predominantly negative attitudes toward the built environment which turn individuals toward nature-seeking.
(DOCX)

S5 Table Pull of the natural environment. Selected comments indicate the positive pull nature exerts on individuals.
(DOCX)

S6 Table Perceptual and material barriers to accessing nature. (DOCX)

S7 Table Physical and structural barriers to accessing nature. (DOCX)

S8 Table Safety and risk concerns as barriers to accessing nature. (DOCX)
