# Supplementary material for: Implications of disparities in social and built environment antecedents to adult nature engagement
Source: PLoS One. 2022 Sep 23;17(9):e0274948. doi: 10.1371/journal.pone.0274948 (PMC9506603; doi:10.1371/journal.pone.0274948)
Supplement: S2 Table — Selected participant comments share how formative places in nature in childhood influenced adult nature engagement. (DOCX) [file pone.0274948.s002.docx]

**S2 Table. Formative places in nature.** Selected participant comments share how formative places in nature in childhood influenced adult nature engagement.

- *I grew up in the mountains outside of Denver. For me, what I think nature is like is an amalgamation of the rivers of Idaho granite, and then the high alpine environment in the Rockies.* Tempe, AZ. Place of origin subtheme
- *I’m from Atlanta. When I think of nature I think of a park in the middle of the city, like Piedmont Park ‘cause I’m from the city, so I always have that connection.* Urban Atlanta. Place of origin subtheme
- *My parents didn't have a ton of money, so we spent most of our vacations going out and camping in the summers. And I remember one year asking my dad if we could move there permanently. So I just fell in love with the outdoors, and I was comfortable.* California. Family Vacation subtheme
- *We grew up on a farm, and that gave you a wonderful appreciation of pastures and soils and growing things. We got dragged to the farm when we were 11 years old to work. We were kids pulling weeds in the garden from the time we could identify a weed from a flower.* Suburban CT. Growing up near nature subtheme
- *The most important aspect of nature for me is the ‘majestic landscape’ that can be seen from the dusky late afternoon. There is nowhere you can’t see it, it’s absolutely breathtaking. I’ve seen beautiful landscapes here in the US, but I will always be a flag-waver, a cultural ambassador for my region, for my beloved Colombia, wherever I go.* Colombia. Uniqueness of own nature subtheme
- *A lot of my conception of nature was shaped kind of oddly by a realization that what I grew up with as nature does not correspond with the rest of the world. And so there is a global nature, but there is also this local nature, and they're part of each other but not the same.* Tempe, AZ. Uniqueness of own nature subtheme
- *I was raised and grew up in a place that's surrounded by national forests. So we had free reign when I was a kid, where we roamed freely wherever we wanted in 15-40,000 acres of forest nearby.* Texas. Unsupervised play in childhood subtheme
- *My girls took their dolls up the hill to the blue dot unmarked trail. And they played in the woods they called ‘The Moss Field.’ I had a bell I’d ring when it was time to come down for dinner. The kids did this, late ‘60s, ‘70s. Today, my girls said they’re not doing that with their kids, my granddaughters.* Suburban CT. Unsupervised play in childhood subtheme
