# Supplementary material for: Implications of disparities in social and built environment antecedents to adult nature engagement
Source: PLoS One. 2022 Sep 23;17(9):e0274948. doi: 10.1371/journal.pone.0274948 (PMC9506603; doi:10.1371/journal.pone.0274948)
Supplement: S3 Table — Selected participant comments discuss formative persons or organizations who socialized them to nature in childhood. (DOCX) [file pone.0274948.s003.docx]

**S3 Table. Formative persons and organizations.** Selected participant comments discuss formative persons or organizations who socialized them to nature in childhood.

- *My Dad was the one who took me outside physically. I probably was in preschool or Kindergarten, too young at the time to know how to get in touch with myself and how to help myself in ways that were needed. Just the act of having a parent physically take me to the natural spaces, to the outdoors and nothing else, just take me there helped.* Berkeley. Parent subtheme
- *My mother was June Cleaver. She stayed at home, but my mother was the active outdoors person who fished. My father was actively outdoors, but only on his job with his geology background…So those were pretty formative years.* Wisconsin. Parent subtheme
- *I say it's my grandfather, without a doubt. Kind of by accident he opened my eyes saying, ‘You know, son, you could have access to it too. It's available to you if you want it.’* Phoenix. Extended family subtheme
- *I love that you use the word ‘cultivation’ toward nature because I feel like my grandfather cultivated this love in me. And when I take out city kids today, they don't even see nature.* Berkeley. Extended family subtheme
- *And at night, when you weren’t camping, they’d build a big bonfire, and this is gonna sound so corny, but I really loved it. There was no other light around - it was pitch black dark and you could see the sky the way you couldn’t in a city - and there was something very grounding about that before you’d go to bed, particularly as a child.* Urban Atlanta. Camp/ counselor subtheme
- *There was one time I went canoeing and I was having a good time, until one of the counselors canoeing with us said to stop canoeing and just lay back and listen to everything. So I did that, and it was really nice. It was like one of those pictures except I was in it.* Urban CT. Camp/counselor subtheme
- *I was in the Scouts as a kid and my family was from Brooklyn so, for them, nature was the rat in the garbage can or the roach running through the kitchen. So, they put me into Scouts when I was a kid, I got camping, and I kind of miss that.* Suburban Atlanta. Scouts/other organization subtheme
- *I remember freshman year I lived in a learning community, and they would have outdoor activities. One of the activities was going to Cascade Falls, which is a natural waterfall, but you had to hike there. That was my first experience hiking, and I really enjoyed it. And from there on, I continued to hike, get that exercise, and integrated it into my daily routine.* Suburban Atlanta. College activity subtheme
- *Yeah, I don’t think I really have anyone in my family in-tune with nature, like no one gardens or anything like that, so I’d say school was my main influence, probably.* Urban CT. School/school activity subtheme
