# Supplementary material for: Implications of disparities in social and built environment antecedents to adult nature engagement
Source: PLoS One. 2022 Sep 23;17(9):e0274948. doi: 10.1371/journal.pone.0274948 (PMC9506603; doi:10.1371/journal.pone.0274948)
Supplement: S4 Table — Selected comments reveal predominantly negative attitudes toward the built environment which turn individuals toward nature-seeking. (DOCX) [file pone.0274948.s004.docx]

**S4 Table. Push of the built environment.** Selected comments reveal predominantly negative attitudes toward the built environment which turn individuals toward nature-seeking.

- *For me, basically anything that’s not surrounded by four walls and fluorescent lighting. If I’m outside, even just sitting on my balcony and being able to see the whole span of Atlanta from my balcony makes me very peaceful.* Suburban Atlanta. Frustration with being indoors subtheme
- *Obviously, anything's better than being in a building, right?* Phoenix. Frustration with being inside subtheme
- *Working in that kind of environment kind of moved me forward til I said, I can't do this, 10 years I can be in that cubicle. Maybe after 5 years I can be moved and look outside this window, look outside my door. I got to be kidding. So I quit my job, went back outdoors.* MN. Frustration with being indoors subtheme
- *Most of the iconic things, that's nature. When you start mixing the human influence, that's when you lose the sense of nature. The more manufactured the thing is, the less it feels like nature.* TX. Human influence in nature subtheme
- *I’m from a very small town in north Georgia and my, I guess “perfect nature” would actually be something like Piedmont Park where it’s really open, and there’s a lot of green space, but you also still have the city and there’s still - there’s not a lot of people, but there’s still people there.* Urban Atlanta. Human influence in nature subtheme
