# Supplementary material for: Implications of disparities in social and built environment antecedents to adult nature engagement
Source: PLoS One. 2022 Sep 23;17(9):e0274948. doi: 10.1371/journal.pone.0274948 (PMC9506603; doi:10.1371/journal.pone.0274948)
Supplement: S5 Table — Selected comments indicate the positive pull nature exerts on individuals. (DOCX) [file pone.0274948.s005.docx]

**S5 Table. Pull of the natural environment.** Selected comments indicate the positive pull nature exerts on individuals.

- *I grew up in Jersey. And my touchpoint is being anywhere in the woods. Just immediately any stress you're feeling or anything that you’re having a hard time with, it just kind of melts away.* Suburban Atlanta. Pull of natural environment subtheme
- *If I crave the water - that’s how I call it, I crave the water - I have to see water, I have to go somewhere. I’ll drive to Acworth and go to the lake up there. Or if I really need to see the ocean, take a long drive to the Gulf, and go see the ocean.* Suburban Atlanta. Pull of natural environment subtheme
- *Outdoors and nature. They both share no walls, no roof. So it gives you the freedom. Me, personally, I like to escape the city, especially if I have a bike or a kayak just so I can go out and see nature. The wide rolling hills, preferably without any manufactured structures, without any built structures.* Phoenix. Nature as place of escape subtheme
- *So if things are overwhelming indoors with people, you know, in indoor spaces, escaping to the outdoors becomes a safe space. And, you know, if I want to feel safe. I think about or go to the outdoors.* Berkeley. Nature as place of escape subtheme
- *Being from NYC, I associated being in nature with an absence of trash. It took a while, two hours to get out of the city to be near nature. Being in Boston, I don’t have to go two hours to get into nature. But my definition of nature has changed since I want more solitude. I want it to be quiet.* Boston. Changing need for nature subtheme
