# Supplementary material for: Implications of disparities in social and built environment antecedents to adult nature engagement
Source: PLoS One. 2022 Sep 23;17(9):e0274948. doi: 10.1371/journal.pone.0274948 (PMC9506603; doi:10.1371/journal.pone.0274948)
Supplement: S6 Table — (DOCX) [file pone.0274948.s006.docx]

**S6 Table. Perceptual and material barriers to accessing nature.**

- *Where I am in my life right now is focusing on work and career in school, and I try and combine those, but I feel like I'm cheating if I'm taking that time away to go outdoors for pleasure. A grim reality, it would be nice to emerge from that.* Berkeley. Perceptual barriers subtheme
- *Personal motivation more than anything. I’m out in the woods all the time, and yet there are times I’m feeling lazy, but I’m out as much as I can.* Suburban CT. Perceptual barriers subtheme
- *When it’s too hot, it depends on the person - those that love it, and those that prefer to stay inside in air conditioning.* Mexico. Perceptual barriers subtheme
- *Kids can’t run, you can only walk on the little path that’s around it. So those are types of restrictions that they have here.* Urban Atlanta. Public and social restrictions subtheme
- *They have certain times that they’re open, you have to plan to be there early so you can be there long enough - if you do have to drive or travel - to make that trip worth it.* Urban Atlanta. Public and social restrictions subtheme
- *For some people, it might be money. ‘Cause there’s some nice parks that you have to pay admission for. Also, whether it be five dollars or twenty dollars, that still may not be money that they have available to them.* Urban CT. Financial resources subtheme
- *Everybody's got to work and nobody’s home to watch the kids. I would love to send my child to walk home. She's eight, going to be nine y/o, and she would enjoy the walk. She loves being outside, but it's the other adults, the nosy parents and neighbors. I'll give her other opportunities to do that, but you know it’s time and money, and it’s hard to get the time off to work to do that.* Phoenix. Perceptual and material barriers subtheme
- *Even not having exposure early on I think is an obstacle. So many kids don't have exposure—we don’t get it until we’re adults… That wasn’t an obstacle for me, I had exposure from some of my first memories. But I work with lots of kids, and all they’ve ever seen is the city. They’ve never been to the river, even though the river is just right there. They’ve never seen a lake, and those to me are obstacles.* Suburban Atlanta. Perceptual and material barriers subtheme
- *Video games, etc. That’s especially true with the younger people. Some of the kids I care for say they don’t want to go outside because they want to play on their iPad. Or they want to watch movies or have their nose in any electronic instead of going outside, starting at three years old. Hallucinating!* Argentina. Technology subtheme
- *Well, I think social media has played a really big impact, because I keep on getting on my phone an ad for ‘Riding the T to a natural destination.’ And I wouldn’t even know that option existed otherwise.*  Boston. Technology subtheme
- *Generationally. Yeah. My 21-year-old son has friends who don't leave their house.* Boston. Generational differences subtheme
