# Supplementary material for: Implications of disparities in social and built environment antecedents to adult nature engagement
Source: PLoS One. 2022 Sep 23;17(9):e0274948. doi: 10.1371/journal.pone.0274948 (PMC9506603; doi:10.1371/journal.pone.0274948)
Supplement: S7 Table — (DOCX) [file pone.0274948.s007.docx]

**S7 Table: Physical and structural barriers to accessing nature.**

- *For me, nature is what I need it to be. So if I go out in my backyard and I need nature, my backyard with the trees and the bird and the squirrels and the possums and raccoons. For me, it doesn’t necessarily have to be going out in the wilderness to find nature.* Suburban Atlanta. Effort to access nature subtheme
- *I mean, it’s pretty easy to access nature, all you’ve gotta do is step outside your house. So it’s very, very effortless, I mean people go outside every day all the time—all you’ve gotta do is throw the trash out and you’re in nature.* Urban CT. Effort to access nature subtheme
- *When I moved to Oakland a little over 20 years ago, I thought where do I go? Where do I find to a hike? And really just having that proximity of the Oakland Hills put me back in a better, healthy place. So very happy to live in Oakland, I love nature… is just… Oh!.* Berkeley. Proximity subtheme
- *Here, I don't feel like I have access to [nature]. I don't own a car. I bike everywhere. I'm always outside. But the nature I want I can't get to. My family still has lots of nature. So it's like living vicariously through their ability to go do things.* Tempe, AZ. Proximity subtheme
- *I am a regional parks ambassador. So I know that there are a lot of trails that are not terribly accessible by public transit. It's insanely hard. Yeah. So if I did not have the transportation I have, I'd be screwed.* Berkeley. Urban infrastructure subtheme
- *So if I’m feeling really stressed out and I’m just going to go for a walk—walk out the front door, walk down the sidewalk, and then come back. Well, if there’s no sidewalk, you kinda can’t walk outside. And if it’s generally an unsafe neighborhood, you’re not going to do that anyways.* Urban Atlanta. Urban infrastructure subtheme
- *If you’re driving somewhere, living in Atlanta, you’re just going to say, ‘Never mind.’ Cause they’ll be like, ‘Oh it’s down the street.’ You’re spending three hours in a car because of traffic. So you’re just like “Never mind, I might as well just stay home.’* Urban Atlanta. Urban infrastructure subtheme
- *I really do think there’s a totally inequity in our system that gets some kids out and others not, and as a result, who’s going to love nature if they don’t have access to it?* Boston. Equity subtheme
