# Supplementary material for: Implications of disparities in social and built environment antecedents to adult nature engagement
Source: PLoS One. 2022 Sep 23;17(9):e0274948. doi: 10.1371/journal.pone.0274948 (PMC9506603; doi:10.1371/journal.pone.0274948)
Supplement: S8 Table — (DOCX) [file pone.0274948.s008.docx]

**S8 Table. Safety and risk concerns as barriers to accessing nature.**

- *I think nature is unsafe, very unsafe, because in order to be safe in nature, you have to come to nature prepared. It can very easily turn lethal.* Tempe, AZ. Risk management subtheme
- *I feel like there is a difference between safety and comfort. I feel like there’s a common thread of something that resonates inside that is calming, it’s a grounding experience when you’re connecting with the outdoors. But safe? I’m not sure it's necessarily safe.* Suburban Atlanta. Safety and risk subtheme
- *So the risk is there. But that's not what I think about. Risk and safety are not the same thing. I wouldn't go there if there's risk.* NH. Safety and risk subtheme
- *The resident bear...poison ivy…ticks…. mosquitos…infectious diseases. I’m a city girl. I only go walking in woods with someone else. If you don’t have a cell phone with you, if you get lost or injured, there’s no one to call. In the city, there’s always someone around to help.* Suburban, CT. Risk management subtheme
- *Since I go out into nature photography and I get alone by myself, I have never felt unsafe in nature. But I have felt unsafe with the people that I have met. And so I actually just took a self-defense class, not against nature, but the people. Yeah, I feel much safer in nature than I do in the city.* Berkeley. Fear of nature vs people subtheme
- *I think it says a lot about the world that I'm far more scared of people than I am of snakes.* Tempe, AZ. Fear of nature vs people subtheme
- *I don’t do well when there aren’t people. We don’t want to be found in the woods.* Urban Atlanta. Fear of nature vs people subtheme
- *I, too, have had experiences not on the trail, but out in suburbia where I was assaulted. And so in my mind, it's safer for me in the outdoors, in nature where I'm, you know, woman vs. mother nature rather than woman vs. humanity.* Berkeley. Women’s safety subtheme
- *I would say that my character has being shaped a lot by my outdoor experiences, including a couple of survival experiences where it wasn't clear that we were going to make it. So we survived, getting stranded out in the middle of nowhere. You learned to push. You've learned that you can do a lot more than you think you can do.* Phoenix. Thrill of pushing safety boundaries subtheme
- *I am very adventurous. When I am very close to an edge, a precipice, I know that a minimal error could cost someone their life. So the risk is more with me, a personal risk, than one that lies intrinsically in nature.* Colombia. Thrill of pushing safety boundaries subtheme
- *You realize that you're so small compared to the scope of things, that you're exposed in a dangerous way to nature, that you're just sort of vulnerable in the landscape.* Tempe, AZ. Vulnerability in nature subtheme
